# The 3D World of Spheroids: Searching for an Optimal Method of Fabricating Pro-Reparative Cardiospheres

**DOI:** 10.3390/ijms262412025

**Published:** 2025-12-13

**Authors:** Yulia Goltseva, Zoya Tsokolaeva, Irina Iarushkina, Irina Beloglazova, Maria Boldyreva, Elizaveta Ratner, Yelena Parfyonova, Konstantin Dergilev

**Affiliations:** 1 Institute of Experimental Cardiology Named after Academician V.N. Smirnov, Federal State Budgetary Institution National Medical Research Center of Cardiology Named after Academician E.I. Chazov, Ministry of Health of the Russian Federation, 121552 Moscow, Russia; ydgoltseva@cardio.ru; 2 Federal Research and Clinical Center of Intensive Care Medicine and Rehabilitology, 141534 Moscow, Russia

**Keywords:** cardiospheres, spheroid, U-well, poly-D-lysine, ultra-low attachment, vascularization, 3D cell culture, angiogenesis

## Abstract

Cardiospheres (CSs) are widely used to boost the pro-reparative potential of adult cardiac cells, mediated through their unique secretome profile. The original CS generation method relies on self-assembly of cardiac explant-derived cells (EDCs) on poly-D-lysine (PDL)-coated plates, but yields inconsistently sized spheroids, restricting broader applications. To address this, we employed ultra-low attachment (ULA) U-well plates to promote uniform spheroid assembly. We systematically compared CSs generated from mouse EDCs using the standard method, based on PDL-coated plates, and the alternative approach, based on ULA U-well plates. Both methods produced viable CSs mimicking the cardiac microenvironment, including mesenchymal cells/fibroblasts, smooth muscle, endothelial, and progenitor cells. PDL-formed CSs were characterized by size heterogeneity, increased stiffness, and reduced endothelial cell content. Despite that, they demonstrated elevated secretion of angiogenesis-related factors and robust proangiogenic potential in vivo. In contrast, generation of mCSs on ULA U-well plates resulted in the formation of soft spheroids with uniform size, enhanced vascularization (CD31+ cells), and increased MCP-1 secretion. In summary, the alternative U-well-based approach enables the generation of uniform spheroids with high spontaneous vascularization, while traditionally formed CSs using PDL-coated plates maintain their superior proangiogenic potential.

## 1. Introduction

Spheroids have emerged as a powerful tool in biomedical research, providing a unique platform for personalized medicine, tissue regeneration, drug development/screening, and basic research. These three-dimensional (3D) culture systems bridge the gap between artificial monolayer cultures and native tissues, where cells are embedded in a specialized microenvironment that maintains tissue homeostasis and function. During 3D cell culturing, cells form a multidimensional network with neighboring cells and surrounding extracellular matrix (ECM) that reproduces physiologically relevant biochemical and mechanical stimuli (e.g., oxygen/nutrient gradient and matrix stiffness). In contrast to two-dimensional (2D) cell cultures, these conditions better preserve the native cell phenotype and viability, providing niche-like support for stem/progenitor cells through mechanisms including secretome modulation and regulation of proliferation/differentiation [1,2,3,4,5]. These advantages underlie the widespread use of 3D cell cultures, including cardiospheres (CSs), to boost cells’ pro-reparative potential [6,7,8]. To achieve this, novel fabrication approaches and modifications are being designed.

CSs are cardiac multicellular spheroids classically generated by culturing cardiac explant-derived cells (EDCs) on poly-D-lysine (PDL)-coated plates [8]. They reproduce a cardiac niche-like microenvironment characterized by core localization of cardiac progenitor cells (CPCs) surrounded by mesenchymal-like and vascular cells [8,9,10]. Their monolayer derivative—cardiosphere-derived cells (CDCs)—is being actively investigated in clinical trials for treating cardiovascular diseases, Duchenne muscular dystrophy, pulmonary hypertension, and COVID-19 [11,12,13,14,15]. Originally developed in 2004 by Giacomello’s research team, CSs enable the derivation of functional CPCs from adult tissues, overcoming previous limitations in cell survival and differentiation capacity for transplantation therapies [8]. Nevertheless, the therapeutic benefit is attributed to robust paracrine activity, rather than direct cell replacement [16]. To date, the gold standard for CS generation remains the self-assembly method based on PDL-coated plates [8]. However, this approach has several limitations, including specialized culture conditions (e.g., growth factor-enriched medium) and high variability in spheroid size distribution.

Multiple modifications of the standard protocol have been attempted to customize CSs and CDCs, and improve their pro-regenerative potential. These include preconditioning under hypoxic or proinflammatory conditions [17,18,19,20], and targeted modulation of key signaling pathways (ERK, β-adrenergic, Notch, and Wnt) [20,21,22,23] to enhance paracrine and proangiogenic activity and optimize CS phenotype. A protocol involving increased cell seeding density and reduced culture duration has been proposed to address the issue of CS size variability [24]. Alternative 3D self-assembly approaches may help overcome the limitations of CS formation on PDL. For instance, hanging drop [7] and ultra-low attachment (ULA) surfaces [4,25] have been proposed for CS assembly. Nevertheless, it remains unclear how the assembly method affects the properties of the resulting CSs and what opportunities exist for spheroid customization. Addressing this question requires systematic side-by-side comparisons of different spheroid assembly approaches.

In this work, we aimed to evaluate an alternative 3D cell assembly platform (96-well ULA plates with U-shaped bottom) against the “gold standard” method based on PDL-coated plates, assessing CS characteristics and proangiogenic capacity. ULA U-well plates were chosen based on their ability to generate uniform spheroids and integrate with high-throughput workflows [26]. To achieve this goal, we formed CSs from mouse EDCs on ULA U-well plates and on PDL-coated plates, and performed comparative analysis. The size, mechanical properties (stiffness), biomarker expression (CD31 and CD73), fibronectin deposition, secretome profile, and in vivo proangiogenic potential of CSs varied significantly depending on the spheroid-generation method employed. We hope that these results will assist researchers in selecting optimal CS assembly approaches tailored to specific objectives.

## 2. Results

### 2.1. The Self-Assembly Process of CSs Demonstrates Distinct Differences Between ULA U-Well Plates and PDL-Coated Plates

**Figure 1:**
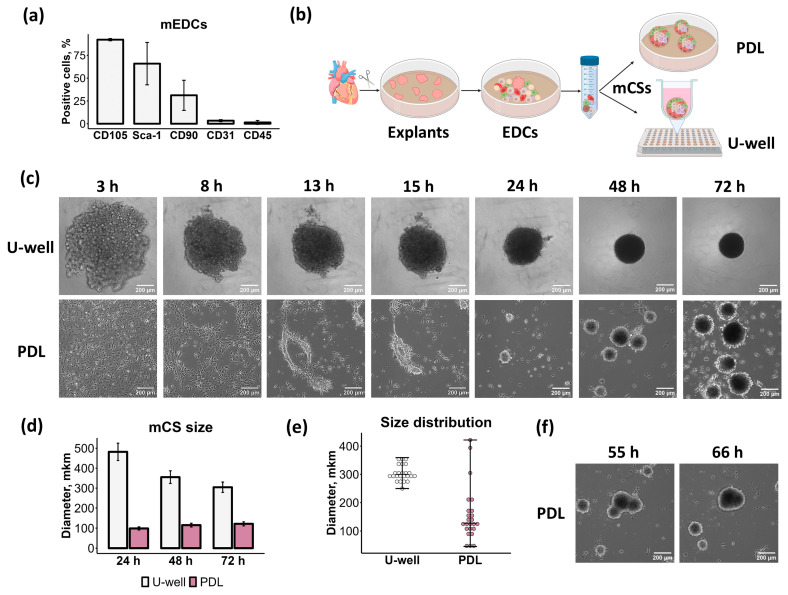
Self-assembly of mouse cardiospheres (mCSs) by two different methods: ultra-low attachment (ULA) U-well plates and poly-D-lysine (PDL)-coated plates with flat bottom. (**a**) Bar plot of flow cytometry analysis of explant-derived cells (EDCs) from mouse hearts. (**b**) The diagram illustrates a methodology for the generation of mCSs. Created in BioRender https://BioRender.com/m27u703 (accessed on 25 July 2025). (**c**) Time-lapse images of mCS formation from EDCs plated on U-well plates and PDL-coated plates. (**d**) Average size of mCSs (mean ± standard deviation), *n* = 3. (**e**) Size distribution of individual mCSs (median ± range). (**f**) Phase-contrast images of mCS fusion.

The original method for CS generation, developed by Giacomello’s research team, relied on spontaneous self-assembly of EDCs on PDL-coated plates using growth factor-enriched medium (Full CS medium) [8]. While this approach has undergone only minor modifications and remains widely used [3,11,12,13,14], its major limitation is the production of inconsistently sized spheroids with variable compactness, restricting broader applications. To address this, we employed ULA U-well plates, which promote uniform spheroid assembly [27,28] and are well-established for generating spheroids from various cell types [26]. This is achieved by assembling one spheroid per well under gravity force, and by preventing any contact between cells and the substrate. Moreover, unlike the traditional PDL-based approach requiring specialized medium supplements, the ULA U-well platform supports CS assembly in basic serum-containing medium (Base CS medium), offering significant advantages for large-scale applications and in vitro modeling. Accordingly, we performed a side-by-side comparison of CS formed in (1) ULA U-well plates using Base CS medium versus (2) PDL-coated plates using Full CS medium. Considering that human and murine CSs have similar composition and properties [29,30,31], we used the EDCs from mouse hearts. The mouse EDCs were positive for CD105 (92%), Sca-1 (66%), CD90 (31%), and CD31 (3%), while they were almost negative for CD45 (<1%) (Figure 1a).

There were significant differences in the process of mouse CS (mCS) formation by EDCs plated on ULA U-well plates and on PDL-coated plates with flat bottom (Figure 1b). In ULA U-well plates, cells interacted with one another to form clusters that subsequently reorganized, progressively compacted, and contracted into 300 μm diameter spheroids (Figure 1c,d, Supplementary Film S1). Conversely, cells in PDL-coated plates adhered to the substrate, formed cell clusters that contracted into spheroids, detached, and grew to 40–400 μm (average 120 μm) through fusion with neighboring spheroids (Figure 1c–f, Supplementary Films S2 and S3). Crucially, beyond differences in cell compaction mechanisms, these two methods demonstrated significantly different spheroid assembly time rates. In ULA U-well plates, cells formed spheroid-like structures within 8 h, whereas in PDL-coated plates, the process was slower, taking 24 h (Figure 1c). At the same time, both types of mCS were recognized as fully formed 72 h after the cells were placed in the wells. The mCSs formed in the ULA U-well plates were uniform in size (Figure 1e) and smooth.

Taken together, both assembly methods rely on distinct cellular self-aggregation mechanisms. ULA U-well plate method produces more uniform CSs compared to the traditional PDL-based approach.

### 2.2. Spheroid Assembly Methods Based on ULA U-Well Plates and PDL-Coated Plates Do Not Affect Cell Viability

**Figure 2:**
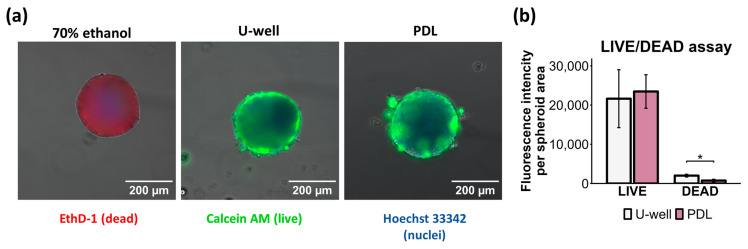
LIVE/DEAD assay of mCSs formed by two different methods: ULA U-well plates and PDL-coated plates with flat bottom. (**a**) Images of mCSs stained by Ethidium Homodimer-1 (EthD-1, dead cells, red), Calcein AM (live cells, green), and Hoechst 33342 (nuclei, blue); mCSs were treated with 70% ethanol as a control for live and dead staining. (**b**) Bar plot of live and dead fluorescence staining signal in mCSs, *n* = 3, * *p* < 0.05.

Cell viability within the spheroid primary depends on its size, which determines the oxygen and nutrient gradient from the external environment to the core [32,33]. Multiple studies have demonstrated that single-cell apoptosis occurs in spheroids of all sizes, while larger spheroids (>500 μm diameter) develop a necrotic core [34,35]. In this regard, we evaluated the viability and cytotoxicity of cells inside mCSs by LIVE/DEAD assay. Although mCSs formed on ULA U-well plates were larger compared to PDL-coated plates, no differences were observed in cell viability, and no necrotic core was observed (Figure 2). Both types of mCSs had very low signal from dead cells in contrast to ethanol-treated mCSs (positive control for dead staining) (Figure 2a). However, a higher signal of dead cell staining was detected within mCSs formed on ULA U-well plates (Figure 2b). This data indicates that both 3D assembly methods preserve high viability of mCSs.

### 2.3. Spheroid Assembly Methods Affect Both the Cellular Composition and ECM Components

**Figure 3:**
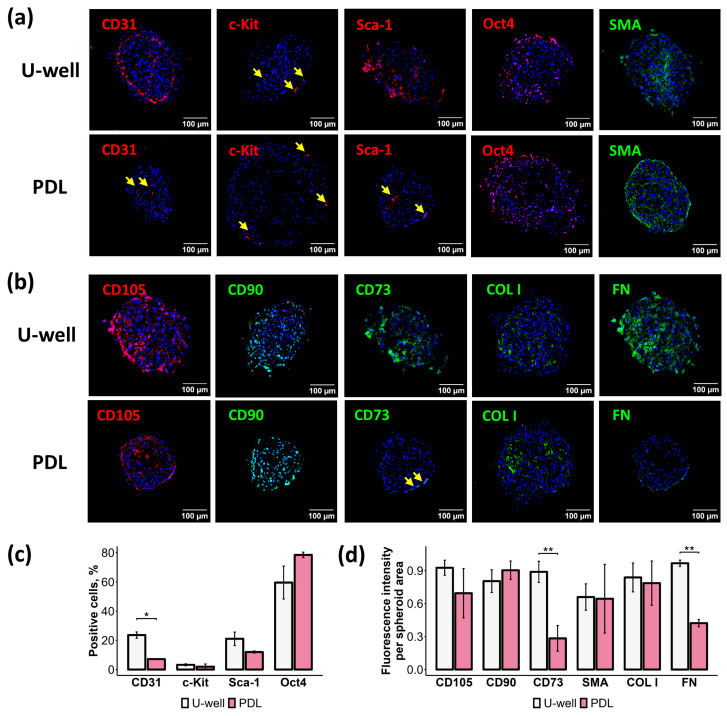
Cell and extracellular matrix (ECM) composition of mCSs formed in ULA U-well plates and PDL-coated plates. (**a**,**b**): Images of mCSs stained by endothelial cell (EC) marker (CD31, red), progenitor cell markers (c-Kit, red; Sca-1, red; Oct4, red), smooth-muscle cell marker (SMA, green), mesenchymal markers (CD105, red; CD90, green; CD73, green), and ECM proteins (COL I, green; FN, green). Nuclei were stained with DAPI. Yellow arrows indicate positive staining. (**c**) Bar plot of a positive staining cell fraction per nuclei within the mCS slice, *n* = 3, * *p* < 0.05. (**d**) Bar plot of fluorescence intensity signal normalized per mCS slice area, *n* = 3, ** *p* < 0.01. SMA—smooth muscle actin, COL I—collagen type I, FN—fibronectin.

We investigated whether the spheroid assembly methods influence their cellular and matrix composition. Vascularization of spheroids is essential for both biofabrication and modeling the capillary-rich cellular microenvironment of the heart [36]. We showed that mCSs on ULA U-well plates contained more CD31-positive endothelial cells (ECs) with no statistically significant differences in vasculogenic c-Kit-positive and Sca-1-positive CPCs [37,38], and Oct4-positive pluripotent-like cells [39] compared to mCSs formed on PDL-coated plates (Figure 3a,c). Furthermore, mCSs on ULA U-well plates were more strongly stained for fibronectin (FN) and CD73 compared to PDL-coated plates, while they had no differences in staining for CD105, CD90, α-smooth muscle actin (SMA), and collagen I (COL I) (Figure 3a,b,d). Despite the observed differences in cellular composition (CD31 and CD73) and fibronectin content, both mCSs contained cardiac perivascular cells and ECM proteins.

### 2.4. CSs Formed on PDL-Coated Plates Demonstrate Enhanced Stiffness Compared to U-Well Plates

**Figure 4:**
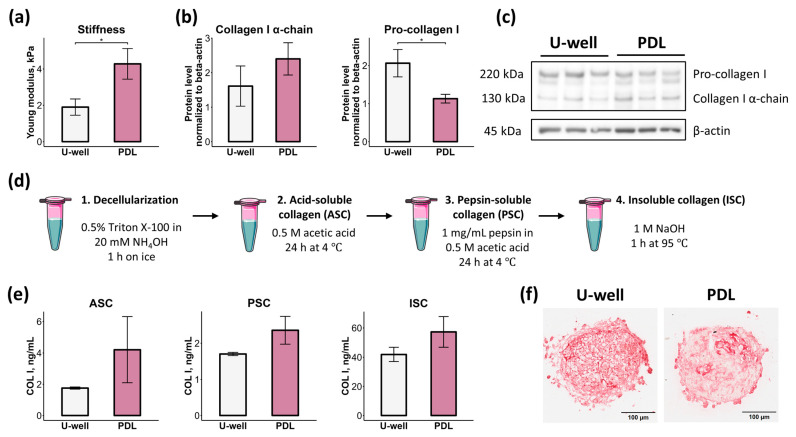
Stiffness and collagen deposition of mCSs formed in ULA U-well plates and PDL-coated plates. (**a**) Young modulus of mCSs, *n* = 3, * *p* < 0.05. (**b**) Bar plots of collagen I α-chain and pro-collagen I protein levels in mCSs, *n* = 3, * *p* < 0.05. (**c**) Western blot images of collagen I and β-actin staining. Original images are presented in Supplementary Figure S1. (**d**) The diagram illustrates a methodology of collagen solubility assay. Created with Servier Medical Art, licensed under CC-BY 4.0. (**e**) Acid-soluble collagen (ASC), pepsin-soluble collagen (PSC), and insoluble collagen (ISC) levels in mCSs, *n* = 3. (**f**) Picro Sirius red staining of mCSs.

Mechanical properties, such as stiffness, significantly contribute to determining spheroid structure and characteristics. Specifically, stiffness modulates cellular sorting within spheroids [40], ECM biosynthesis [41], and the cell secretome [42]. To assess stiffness, we measured the Young modulus of size-matched mCSs using a parallel-plate compression test. mCSs formed on PDL-coated plates demonstrated higher values of Young modulus compared to mCSs formed on ULA U-well plates (Figure 4a), indicating their stiffness.

Next, we assessed collagen I deposition within mCSs since collagen I was shown to promote spheroid stiffness [43]. Similarly to the immunofluorescence data (Figure 3b,d), we did not observe any significant difference in the level of mature collagen I α-chain (Figure 4b,c). However, the level of pro-collagen I was higher in mCSs formed on ULA U-well plates compared to those formed on PDL-coated plates (Figure 4b,c).

Collagen cross-linking significantly contributes to the mechanical stiffness [44]. We performed a collagen solubility assay to extract soluble and insoluble collagen fractions from mCSs (Figure 4d). Acid-soluble collagen (ASC) corresponds to newly synthesized collagen, pepsin-soluble collagen (PSC) represents a more stable/mature fraction, while insoluble collagen (ISC) consists of highly cross-linked collagen [45]. mCSs formed on PDL-coated plates showed non-significant increases in ASC, PSC, and ISC content versus mCSs formed on ULA U-well plates (Figure 4e), possibly reflecting enhanced stiffness. Moreover, Picro Sirius Red staining of fibrillar collagens revealed that collagen distribution in PDL-formed mCSs appeared more compact compared to the more dispersed organization observed in ULA U-well spheroids (Figure 4f).

**Figure 5:**
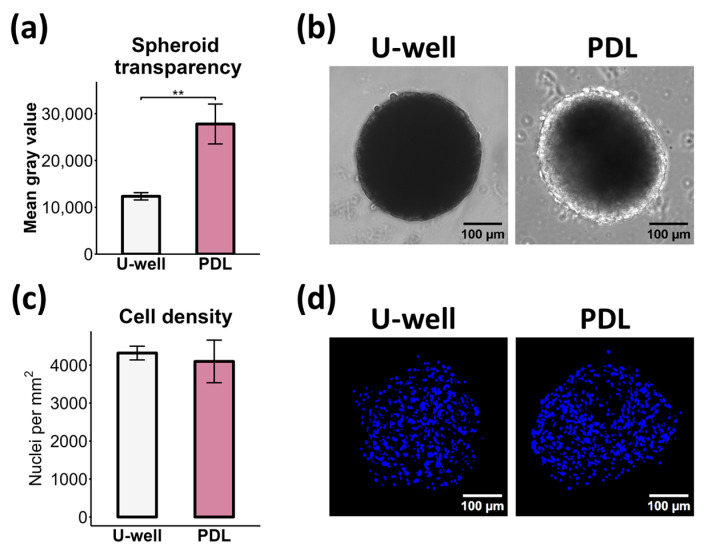
Transparency and cell density of mCSs formed in ULA U-well plates and PDL-coated plates. (**a**) Bar plot of spheroid transparency measured as mean gray value, *n* = 3, ** *p* < 0.01. (**b**) Phase-contrast images of mCSs. (**c**) Bar plot of cell density within mCSs measured as nuclei number per area, *n* = 3. (**d**) Immunofluorescence images of nuclei (DAPI) staining of mCS cryosections.

We noticed that size-matched (equal-sized) mCSs formed on PDL-coated plates were more transparent compared to mCSs formed on ULA U-well plates (Figure 5a,b), suggesting distinct ECM density or cellular packing [46,47]. However, we found no significant differences in cell density between size-matched mCSs formed on ULA U-well plates and PDL-coated plates (Figure 5c,d), in agreement with other studies [48]. Moreover, even after decellularization, spheroids maintained their low level of transparency (Supplementary Figure S2). These findings suggest that spheroid transparency may be associated with overall ECM deposition and organization (Figure 4f) rather than cell density.

Collectively, these data indicate that formation of mCSs on PDL-coated plates resulted in the generation of stiffer spheroids with high ECM density.

### 2.5. Spheroid-Forming Methods Significantly Influence the Secretome Profile of CSs

**Figure 6:**
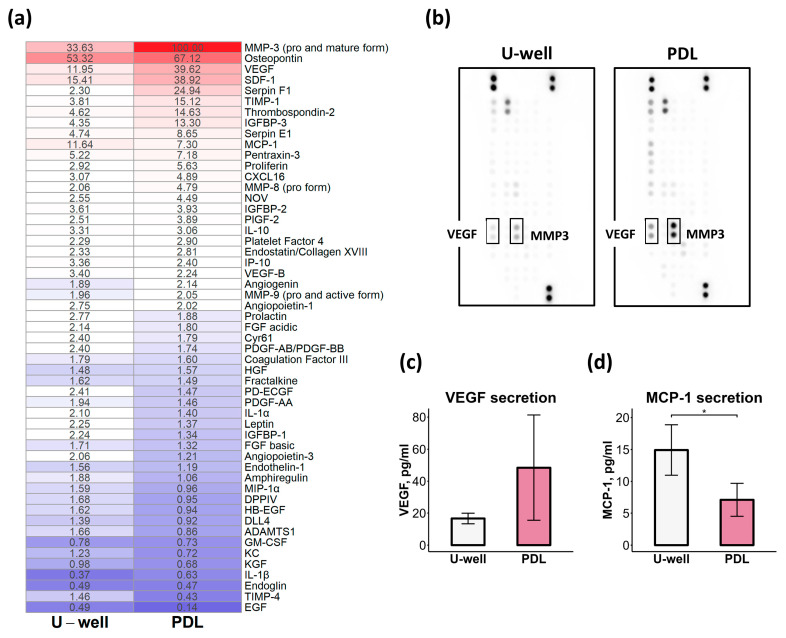
Profile of angiogenesis-related proteins secreted by mCSs formed in ULA U-well plates and PDL-coated plates. (**a**) A heatmap of the secreted factor levels in conditioned media of mCSs measured by Proteome Profiler^TM^ Mouse Angiogenesis Array Kit (R&D). The color gradient represents relative concentration levels, with blue indicating low and red indicating high concentrations. (**b**) Array images of angiogenesis-related protein staining. Bar plots of VEGF-A (**c**) and MCP-1 (**d**) levels in mCS condition mediums measured by ELISA, *n* = 3, * *p* < 0.05.

Given the significant differences in self-assembly process, size distribution, and cellular/ECM composition between mCSs formed on ULA U-well plates and PDL-coated plates, we hypothesized that these variations would translate to distinct secretome profiles. We analyzed a profile of secreted angiogenesis-related proteins in condition media of mCSs by Mouse Angiogenesis Array Kit (Figure 6a,b).

Some proangiogenic factors, including metalloproteinase-3 (MMP-3), osteopontin, vascular endothelial growth factor (VEGF), stromal cell-derived factor-1 (SDF-1), and monocyte chemoattractant protein-1 (MCP-1), were enriched in the conditioned media of both mCS types. The secretome of mCSs formed on ULA U-well plates showed consistent secretion levels (~2 units) for most analyzed angiogenesis-related proteins (Figure 6a,b). mCSs formed on PDL-coated plates demonstrated significantly altered secretome profile versus mCSs formed on ULA U-well plates, with upregulated secretion of both proangiogenic (MMP-3, VEGF, SDF-1, IGFBP-3) and anti-angiogenic factors (Serpin F1, TIMP-1, thrombospondin-2) (Figure 6a,b). Only MCP-1, one of the proangiogenic factors, was found to be elevated in the conditioned media of mCSs formed on ULA U-well plates, which was also confirmed by ELISA (Figure 6a,d). While mCSs in ULA U-well plates maintained uniform VEGF secretion profiles, mCSs formed on PDL-coated plates showed significant sample-to-sample variability (Figure 6c). This variability likely arises from the heterogeneous size distribution of mCSs formed on PDL-coated plates.

Collectively, the PDL-based 3D assembly approach enhances the secretome profile of mCSs, characterized by increased production of both proangiogenic and anti-angiogenic factors compared to ULA U-well plates.

### 2.6. Both CS Culture Medium and Substrate Affect mCS Properties

To investigate the effect of culture medium on mCS properties, we formed mCSs on ULA U-well plates both in Base CS medium (supplemented with only 3% serum) and Full CS medium (supplemented with 3% serum, EGF, bFGF, cardiotrophin-1, thrombin, and NeuroBrew-21 without vitamin A). Notably, while EDCs form spheroids on PDL-coated plates exclusively in Full CS medium, they remain as a monolayer in Base CS medium.

**Figure 7:**
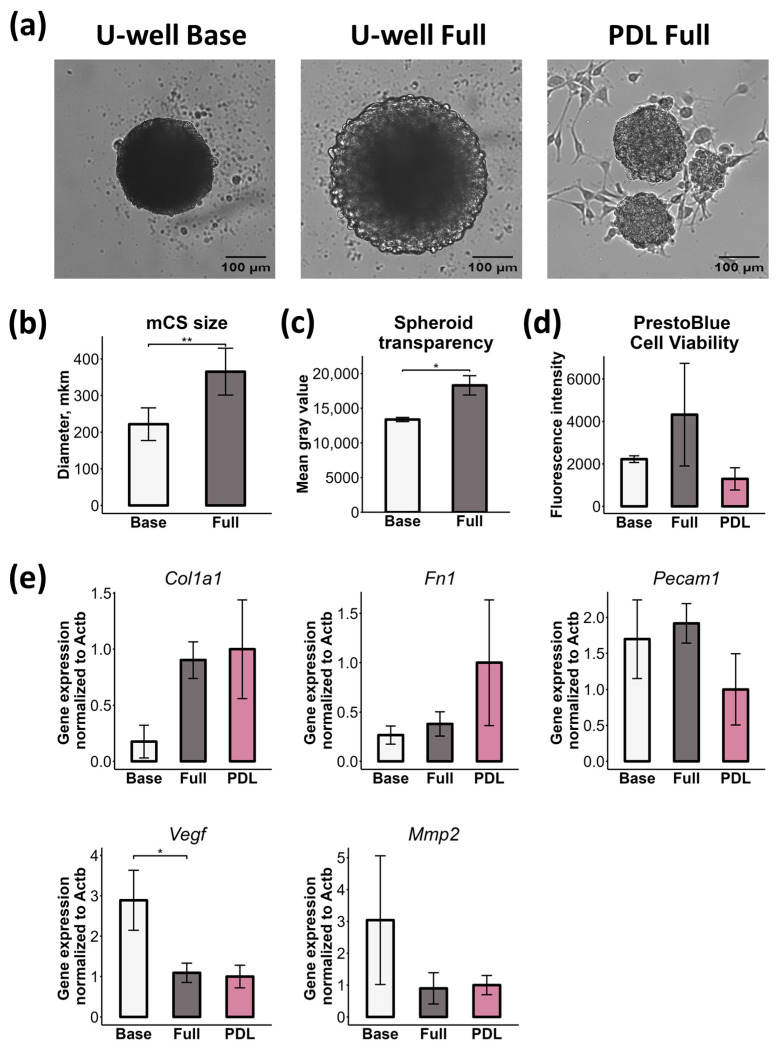
Culture medium and substrate effects on mCS morphology and gene expression. (**a**) Brightfield images of mCSs formed on ULA U-well plates in Base CS medium (Base), ULA U-well plates in Full CS medium (Full), and on PDL-coated plates in Full CS medium (PDL). (**b**) Average size of mCSs formed on ULA U-well plates in Base or Full CS medium, *n* = 3, ** *p* < 0.01. (**c**) Bar plot of spheroid transparency measured as mean gray value, *n* = 3, * *p* < 0.05. (**d**) Bar plot of mCS cell viability measured with PrestoBlue, *n* = 3. (**e**) Bar plots of *Col1a1*, *Fn1*, *Pecam1*, *Vegf*, and *Mmp2* gene expression levels in mCSs, *n* = 3, * *p* < 0.05.

Culturing mCSs on ULA U-well plates in Full CS medium resulted in the formation of large spheroids with greater transparency and a rougher surface morphology compared to those formed in Base CS medium (Figure 7a–c). These large mCSs formed in Full CS medium on ULA U-well plates exhibited a higher (though non-significant) viability rate than those formed in Base CS medium, as was assessed by PrestoBlue assay (analog of MTT assay) (Figure 7d). We suppose that growth factors from Full CS medium (such as EGF and bFGF) form the gradient within the spheroids, thereby driving the observed proliferation in the outer layers of mCSs. We also assessed the viability of mCSs formed on PDL-coated plates using the PrestoBlue reagent. Consistent with the LIVE/DEAD assay results (Figure 2), the viability of mCSs formed on PDL-coated plates was comparable to that of mCSs formed on ULA U-well plates (Figure 7d). The slight non-significant reduction in viability rate of PDL-derived spheroids may be associated with their smaller diameter relative to mCSs formed in ULA U-well plates (Figure 1e and Figure 7a).

As demonstrated above, mCSs formed on ULA U-well plates differ from those generated on PDL in several aspects, including (1) stiffness, (2) deposition and distribution of ECM proteins, (3) cellular composition (CD31 and CD73 expression), and (4) secretory activity. To investigate the contributions of culture medium and substrate/plate type, we therefore systematically evaluated the effects of both CS medium (Base vs. Full) and assembly approach (ULA U-well vs. PDL) on the gene expression of matrix components, EC marker, and proangiogenic genes. A trend toward higher *Col1a1* (collagen type I) expression was observed in mCSs formed on ULA U-well plates in Full CS medium compared to those formed in Base CS medium, and no significant differences in *Col1a1* expression were detected between mCSs generated using different approaches (ULA U-well vs. PDL, both in Full CS medium) (Figure 7e). This finding aligns with our collagen deposition data obtained through the collagen solubility assay (Figure 4e).

For *Fn1* (fibronectin), we observed the opposite pattern: the culture medium had minimal effect on its expression, whereas substrate type substantially influenced it (Figure 7e). Specifically, mCSs expressed higher *Fn1* levels on PDL than on ULA U-well plates, although this difference did not reach statistical significance. These results contrast with fibronectin content data obtained by immunofluorescence staining, where mCSs formed in ULA U-well plates exhibited elevated fibronectin levels (Figure 3b,d). This apparent discrepancy may occur from differences in the spheroid assembly process (see Figure 1c and Supplementary Films S1–S3). In ULA U-well plates, cells undergo rapid cell–cell aggregation and immediate 3D structure formation, thereby initiating early fibronectin deposition within the spheroid. Indeed, it was reported that FN expression is induced during the initial spheroid formation in ULA U-well plates and is required for their compaction [49]. In contrast, on PDL, cells remain as a monolayer for approximately 10 h before gradually assembling into spheroids, potentially delaying and altering FN organization.

mCSs formed on ULA U-well plates demonstrated higher vascularization (i.e., CD31+ EC content) compared to PDL (Figure 3a,c). This effect was likely arisen from the assembly approach used to generate spheroids. Thus, a trend toward lower *Pecam1* (CD31) expression was observed for mCSs formed on PDL compared to mCSs formed on ULA U-well (both in Full CS medium) (Figure 7e). The culture medium (Base vs. Full) did not affect *Pecam1* expression.

The expression of proangiogenic factors (*Vegf* and *Mmp2*) was primarily dependent on the culture medium. On ULA U-well plates, *Vegf* expression was significantly higher when mCSs were cultured in Base CS medium compared to Full CS medium (Figure 7e). This differential expression was independent of the assembly approach, as *Vegf* levels did not vary between ULA U-well and PDL-coated plates. A similar medium-dependent effect was observed for *Mmp2* expression (Figure 7e). Although this finding appears to contradict our secretome analysis (dot blot array and ELISA), which showed higher VEGF secretion by mCSs formed on PDL, the discrepancy is readily explainable. We had previously demonstrated that during co-culture of mesenchymal stromal cells (MSCs) with ECs, secreted VEGF protein level decreases despite high mRNA expression, due to its active uptake and utilization by ECs [50].

Taken together, these data demonstrate that both the culture medium and the assembly approach influence spheroid morphology and properties. The CS medium (Base vs. Full) exerts a greater influence on spheroid morphology and expression of *Col1a1*, *Vegf,* and *Mmp2*, whereas substrate/plate type (ULA U-well vs. PDL) regulates *Fn1* and *Pecam1* expression.

### 2.7. CSs Formed on PDL-Coated Plates Stimulate Angiogenesis After Transplantation In Vivo

**Figure 8:**
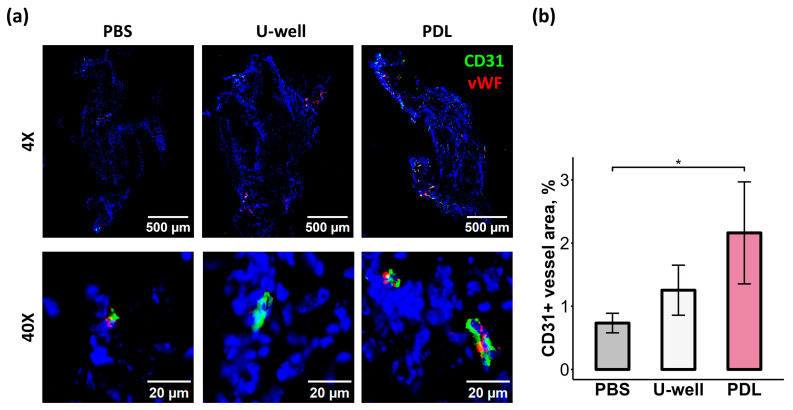
In vivo proangiogenic properties of mCSs formed in ULA U-well plates and PDL-coated plates. (**a**) Immunofluorescence images of cryosections of Matrigel^TM^ loaded with PBS (phosphate-buffered saline, negative control) or mCSs stained for CD31 (green) and vWF (red) after 14 days post-subcutaneous transplantation. The images were magnified to 4× and 40×. Nuclei were stained with DAPI. (**b**) Bar plot of a CD31-positive vessel area per Matrigel area, *n* = 4, * *p* < 0.05.

To determine whether the spheroid-assembly methods affect their proangiogenic properties, we performed subcutaneous transplantation of mCSs formed on ULA U-well plates (in Base CS medium) and PDL-coated plates (in Full CS medium) using a Matrigel plug assay with growth factor-reduced Matrigel. Fourteen days after transplantation, the mCS grafts demonstrated the presence of CD31- and vWF-positive structures (Figure 8a). Compared to the negative control (phosphate-buffered saline (PBS)-loaded Matrigel), mCSs formed on PDL-coated plates significantly enhanced vascular structure formation (three-fold higher), as quantified by the percentage of CD31-positive stained area (Figure 8b). However, no statistically significant differences in vascular structures content were observed between the two types of mCSs. Considering that mCSs formed on PDL-coated plates exhibited lower CD31+ EC contents (Figure 3a,c), we suppose that in vivo vascularization occurs primarily via host vessel incorporation into the Matrigel plug.

## 3. Discussion

CSs are widely used to boost the reparative potential of adult non-myocyte cardiac cells [6,7,8]. While PDL-coated plates remain the most widely used platform for CS production, their limitations in scalability and reproducibility constrain therapeutic translation and large-scale screening. To overcome this, alternative 3D assembly approaches have been undertaken, including the use of ULA U-well plates [4]. In this work, we compared how two CS assembly methods—ULA 96-well plates with U-shaped bottom and PDL-coated well plates with flat bottom—influence CS characteristics and proangiogenic capacity. Our results demonstrate that both methods have their own strengths and weaknesses. The main findings indicate that ULA U-well plates generate more uniform CSs with greater vascularization (i.e., CD31+ EC content) and reduced stiffness, whereas PDL-coated plates produce CSs exhibiting enhanced proangiogenic potential after in vivo transplantation.

Classically, CSs are defined as cardiac spheroids formed from cardiac explant outgrowth (i.e., EDCs) that provide a heterogeneous cell pool (Figure 1a) containing stromal (mesenchymal-like), vascular (smooth-muscle and endothelial cells), and progenitor cell populations, making them an ideal source for modeling the cardiac microenvironment, as demonstrated in this work (Figure 3) and previous studies [4,8,31,51]. Originally developed in 2004 [8], CS were designed to expand CPCs, though later studies revealed their therapeutic benefits are mediated primarily through paracrine effects [16]. Indeed, CSs demonstrate significantly higher production of proangiogenic factors (e.g., VEGF, HGF, MMP-3, and MMP-9) compared to 2D cultures [3,16]. Furthermore, compared to other stromal cell types, such as fibroblasts and MSCs, the secretome profile of CS monolayer derivatives (CDCs) was well balanced with high extracellular vesicle output [16,52,53]. These advantages have established CSs and CDCs as widely used tools in biomedical research.

While PDL-coated plates remain the gold standard method for CS generation, at least two ULA-based alternatives have emerged to address limitations in scalability and reproducibility [4,25]. The mechanobiological mechanisms of spheroid assembly diverge significantly between these substrates (Figure 1c and Supplementary Films S1–S3). PDL provides electrostatic cell–substrate interactions that enable cells to initially adhere and spread before engaging in robust cell–cell and cell–ECM contacts. Spheroid formation on PDL requires specific culture conditions, including high seeding densities and growth factor-enriched culture medium [31,54,55]. These conditions promote integrin-mediated mechanotransduction and activation of intracellular signaling pathways (i.e., MAPK/ERK), leading to cell contraction, “tightening” into 3D aggregates, and modulating cell phenotype and properties [20,56,57,58]. Indeed, applying the growth factor-enriched medium (Full CS medium) for spheroid generation revealed that these conditions induce *Col1a1* expression while reducing *Vegf* and *Mmp2* expression compared to basic medium (Base CS medium) (Figure 7e). On ULA surfaces, cells are forced to interact with each other through the formation of cell adhesion contacts (i.e., cadherins), leading to spontaneous and rapid 3D assembly to minimize free energy [56,59]. Our findings establish that the choice of 3D assembly method critically determines CS composition, immunophenotype, mechanical properties, secretome profile, and proangiogenic potential (Figure 3, Figure 4, Figure 5, Figure 6, Figure 7 and Figure 8).

Generation of mCSs on ULA U-well plates significantly increased the content of endothelial cells (23.6 ± 2.1%) compared to both the original 2D culture of mEDCs (3.4 ± 1.2%) and mCSs formed on PDL-coated plates (7.2 ± 0.2%) (Figure 1a and Figure 3c). It was reported that spheroid cultures induce spontaneous endothelial differentiation of stromal cells [60]. We demonstrated that *Pecam1* expression primarily depends on the 3D assembly approach (Figure 7e). The underlying mechanism could involve the formation of a hypoxic microenvironment, upregulation of proangiogenic factor secretion, and activation of cellular reprogramming [61,62,63]. CD31-positive cells were predominantly localized to the oxygen-rich outer layers of mCSs (Figure 3a), indicating limited hypoxic influence on vascularization. Despite that, we observed increased *Vegf* gene expression (Figure 7e) with decreased secreted VEGF content (Figure 6) in mCSs formed on ULA U-well plates compared to those on PDL. This result suggests that during formation of mCSs on ULA U-well, elevated *Vegf* expression may support endothelial differentiation, leading to increased VEGF protein uptake and a consequent reduction in its concentration in the conditioned media [50].

These effects may be partially attributed to alterations in spheroid mechanical properties, which can influence cellular behavior. We revealed that both types of mCSs can be described as relatively soft: 2 kPa for mCSs on ULA U-well plates and 4 kPa for mCSs on PDL-coated plates (Figure 4a) compared to ~10–40 kPa for myocardium [64,65,66]. Notably, mCSs formed on PDL-coated plates were stiffer compared to ULA U-well plates, which could additionally explain decreased vascularization and enhanced secretory activity [42,67].

Multiple mechanisms may be involved in increased stiffness, including deposition of fibrillar collagens and proteoglycans [68], collagen cross-linking [44] and glycation [69], as well as increased cell contraction [70]. We observed differences in spheroid transparency (Figure 5a,b), suggesting increased overall ECM deposition or distinct ECM organization within mCSs formed on PDL-coated plates [46,47]. On tumor spheroid model, it has been shown that the spheroid-generation method affects collagen I content [71]. We revealed a non-significant trend toward increased newly synthesized collagen I (ASC fraction), mature collagen I (both PSC and collagen I α-chain), and cross-linked collagen (ISC fraction) production in mCSs formed on PDL-coated plates, whereas a higher level of pro-collagen I was observed in mCSs formed on ULA U-well plates (Figure 4a–e). Furthermore, more compact fibrillar collagen organization was observed within mCSs formed on PDL-coated plates (Figure 4f). However, this increased collagen production was possibly induced by culture conditions (i.e., Full CS medium) rather than the substrate type (Figure 7e). Together with compact fibrillar collagen organization, these results could explain the enhanced stiffness of mCSs formed on PDL-coated plates.

Fibronectin is essential for both ECM organization [72] and vascular morphogenesis [73]. We found significantly higher fibronectin deposition within mCSs formed on ULA U-well plates compared to mCSs formed on PDL-coated plates (Figure 3d). Several studies reported that different methods of spheroid assembly (i.e., 3D hydrogel, microwells, and suspension) and different substrates (hydrophilic polymer polyHEMA and more hydrophobic polyCHMA) affect fibronectin expression [74,75]. Our findings (Figure 7e) align with the previous reports. Fibronectin expression could be upregulated by hypoxia, which is increased with spheroid size [61], and by secreted proteins such as TGFβ and endothelin-1 [76,77,78] or downregulated by SDF-1 [79]. mCSs formed on ULA U-well plates secreted higher levels of endothelin-1 and lower levels of SDF-1 compared to mCSs formed on PDL-coated plates (Figure 6a), suggesting secretory-based regulation of fibronectin expression. Nevertheless, precise determination of the mechanism by which spheroid assembly methods affect ECM deposition and stiffness requires further investigation.

Matrix stiffness was shown to regulate cell sorting within spheroids [40]. We observed distinct spatial distribution patterns of SMA-positive cells enriched in the outer layers of mCSs formed on PDL-coated plates versus diffuse, centralized distribution within mCSs formed on ULA U-well plates (Figure 3a). This patterning may potentiate spheroid stiffness through actomyosin-mediated cellular contraction [80]. Another notable evidence was observed regarding CD73 expression, which increased within mCSs formed on ULA U-well plates compared to PDL-coated plates (Figure 3d). CD73 is a membrane receptor and an ecto-5′-nucleotidase that hydrolyses adenosine monophosphate (AMP) to adenosine, which exhibits immunosuppressive and anti-fibrotic properties [81,82,83]. CD73 expression has been shown to enhance ECM-related gene expression and improve the engraftment potential of spheroids in vivo [84], while suppressing myogenic differentiation [85]. We suppose that in mCSs formed on ULA U-well plates, CD73 expression could be induced by hypoxia [86] and associated with ECM deposition.

Our study has several limitations regarding the mechanisms by which the CS assembly approach influences their properties. Our data primarily reveal correlations rather than establishing a direct causal link between factors such as matrix stiffness, fibronectin content, or CD73 expression and the observed capillary-like network formation within mCSs or the regulation of their proangiogenic capacity. Addressing this question definitively will require additional investigations, such as targeted inhibition or comparison of spheroids at different formation stages. Furthermore, this work is a proof-of-concept comparison in a murine model. Further validation of our findings using human-derived cells is required.

Collectively, we demonstrate that the 3D assembly method, as well as culture medium, determines cellular immunophenotype, ECM composition, secretory profile, and spheroid stiffness and vascularization. These modifications may critically contribute to the regenerative capacity of CSs. Using a Matrigel plug assay in a murine subcutaneous transplantation model, we demonstrated that mCSs formed on PDL-coated plates significantly enhanced in vivo angiogenesis, whereas mCSs formed on ULA U-well plates (in Base CS medium) showed only a non-significant trend (Figure 8). It was reported that the regenerative potential of CSs depends on their secretory profile [16]. Indeed, our findings demonstrated that mCS formation on PDL-coated plates enhanced secretion of angiogenesis-related factors, including MMP-3, VEGF, and SDF-1 (Figure 6). We suppose that the proangiogenic potential of mCSs formed on PDL-coated plates reflects their improved secretome.

In summary, both 3D assembly approaches for CS generation—ULA U-well plates and PDL-coated plates—have their own strengths and weaknesses. Although our study is based on murine cells, it is important to note that mouse and human CSs share structural and functional homology [29,30,31]. This supports the cautious extrapolation of the translational potential of our findings. The advantage of the PDL-based method consists of improved secretory profile and proangiogenic potential, which form the basis for their therapeutic application [11,12,13,14]. In addition, on PDL-coated plates, spheroids spontaneously fuse with other ones (Figure 1f), which is necessary to form tissue-like structures [87,88]. However, the complexity of this system and the variability in spheroid size (Figure 1e) limit the application of CSs formed on PDL-coated plates in pharmacological studies and bioprinting techniques that require uniform “blocks”. Nevertheless, researchers attempted to regulate the size [24], phenotype, and secretory profile of CSs [20,21,89] through modifications of the basic protocol. Of note, some bioprinting techniques, like aspiration-assisted bioprinting, are suitable for spheroids with variability in size and shape [90]. Key benefits of the ULA U-well-based method include (1) formation of a uniform-sized single spheroid per well (Figure 1e) and (2) spontaneous vascularization (Figure 3a), which is important for both biofabrication [91] and cardiac vascular niche modeling [4]. Moreover, using ULA U-well plates, spheroid size could be controlled simply by varying the number of seeded cells [32]. Precise control over spheroid size is important for high-throughput screening [92], pathological modeling [35,93], drug screening [94], and advanced bioprinting technologies like the Kenzan method (3D bioprinting on a needle array) [95]. The main disadvantage of the ULA U-well-based method consists of the labor-intensive manual harvesting of spheroids, which limits scalability.

## 4. Materials and Methods

### 4.1. Animals

Adult male C57BL6/129 mice (*n* = 15) were purchased from the National Medical Research Center of Cardiology nursery. All mice were six to eight weeks old (18–22 g). Animals were housed humanely with free access to food and water, and maintained under a 12 h light/dark cycle. Mice were anesthetized before euthanasia with Isoflurane and then cervical dislocation.

### 4.2. Culture of Explant-Derived Cells

To form CSs, we isolated a heterogeneous population of cardiac cells from mouse hearts as described previously [4,8]. Mouse hearts (*n* = 9) were extracted in Krebs-Ringer (Sigma, St. Louis, MO, USA) buffer supplemented with 11.25 U/mL heparin, minced into small pieces (1–2 mm), washed with Ca^2+^, Mg^2+^-free Dulbecco’s Phosphate-Buffered Saline (DPBS), and incubated with a 0.1 mg/mL collagenase A solution (Roche) for 10 min at 37 °C in rotation. To stop the reaction, 10% of fetal bovine serum (FSB, HyClone, Cytivia, Marlborough, MA, USA) was added. The isolated tissue samples (explants) were placed onto 150 mm Petri dishes (one heart per dish) that had been coated with 40 μg/mL fibronectin (Imtek, Moscow, Russia). Cardiac explants were cultured for 10–14 days at 37 °C in 5% CO_2_ in Explant medium based on IMDM (Servicebio, Wuhan, China) supplemented with 20% FBS (Gibco, Waltham, MA, USA), 0.1 mM β-mercaptoethanol (Sigma), and 100 U/mL penicillin-streptomycin (Gibco). Cells that migrated from the explants—explant-derived cells (EDCs)—were harvested by a two-minute incubation with a 0.25% trypsin solution (Gibco) and inactivated by the addition of 10% FBS. EDCs were filtered through a 100 μm cell strainer (BD) and seeded on fibronectin-coated dishes in Explant medium. EDCs were passaged no more than five times. The culture medium was changed every 72 h.

### 4.3. Cardiosphere Formation

We followed two different protocols to form mCSs from EDCs. The first method includes the formation of spheroids on ULA U-well plates [4], while the second one is based on PDL-coated plates with flat-bottom [3,8].

EDCs were harvested by 0.025% trypsin solution (Gibco) followed by inactivation with 10% FBS. EDCs were seeded at 10,000 cells per well in ULA 96-well plates with U-bottom (SPL) with 200 μL Base CS medium consisting of 65% DMEM/F-12 (Gibco), 35% IMDM (Servicebio), 3% FBS (Gibco), and 100 U/mL penicillin–streptomycin (Gibco). Additionally, EDCs were seeded in Full CS medium consisting of Base CS medium supplemented with 20 ng/mL EGF (Paneco, Moscow, Russia), 20 ng/mL bFGF (ProSpec-Tany TechnoGene Ltd., Rehovot, Israel), 8 ng/mL cardiotrophin-1 (PeproTech, London, UK), 1 U/mL thrombin (Sigma), 1× NeuroBrew-21 w/o Vit. A (Miltenyi Biotec, Bergisch Gladbach, Germany). Plates were immediately centrifuged at 400× *g* for 5 min to aggregate cells into the center of a well.

In parallel, EDCs were seeded at 60,000 cells per cm^2^ in PDL-coated 12-well plates (Corning, Corning, NY, USA) with 1 mL Full CS medium.

mCSs were cultured for 72 h at 37 °C with 5% CO_2_ without medium replacement. The process of mCS formation was visualized using an Image Exfluorer AI microscope (LCI, Namyangju-si, Republic of Korea) with a life-maintaining module (37 °C, 5% CO_2_), allowing for taking time-lapse series of images with 20 min intervals. Brightfield images were acquired using a JuliStage live-cell analysis system (NanoEntek, Seoul, Republic of Korea).

### 4.4. Flow Cytometry

The EDCs were trypsinized, washed in ice-cold FASC buffer (5% FBS, 0.1% sodium azide in Ca^2+^, Mg^2+^-free DPBS), and incubated with primary antibodies to CD105 (#120404, Biolegend, San Diego, CA, USA), Sca-1 conjugated with PE/Cy7 (E-AB-F1191UH, Elabscience, Wuhan, China), CD90 conjugated with FITC (E-AB-F1283UC, Elabscience), CD31 (#553370; BD, San Diego, CA, USA), and CD45 conjugated with PE (E-AB-F1136UD, Elabscience) or with isotype IgGs for 30 min on ice. The cells were washed with FACS buffer twice and incubated with the secondary fluorochrome-labeled antibody Goat anti-Rat IgG (H+L) Cross-Adsorbed, Alexa Fluor™ 488 (A11006, Invitrogen) for 30 min on ice when indirect staining was performed. The cells were washed twice and fixed in 1% formaldehyde solution for 10 min with washing. Unstained cells followed the same procedure. The cells were analyzed with a FACSAria III cell sorter (BD) and FACSDiva software (v. 9.0.1, BD).

### 4.5. mCS Viability Assay

To analyze cell viability and cell death within mCSs, we used LIVE/DEAD™ Viability/Cytotoxicity Kit, for mammalian cells (Invitrogen, Carlsbad, CA, USA). mCSs were incubated with 2.5 μg/mL Hoechst 33342 (Invitrogen), 2 μM Calcein-AM, and 4 μM ethidium homodimer-1 for 3 h at 37 °C in the dark. To avoid washing out the spheroids, we used 11× staining mix based on IMDM medium (Servicebio) without a washing step or medium replacement [96]. Few spheroids were pre-incubated with 70% ethanol for 30 min to control live and dead staining. After 3 h of staining, mCSs were collected in 15 mL tubes, centrifuged at 50× *g* for 3 min, and placed on a plate with a glass bottom (Nunc, ThermoFisher Scientific, Waltham, MA, USA) in a transparent medium RPMI (Gibco) supplemented with 3% FBS (Gibco). The fluorescence was visualized using an Image Exfluorer AI microscope (LCI) with a life-maintaining module (37 °C, 5% CO_2_).

Additionally, PrestoBlue viability/metabolic activity assay was performed [97]. Individual mCSs were placed into a flat-bottom 96-well plate (Corning), one spheroid per well in 90 μL Base CS medium. Then, 10 μL PrestoBlue™ Cell Viability Reagent (Invitrogen) was added. Spheroids were incubated with the reagent for 4 h at 37 °C. The fluorometry was assessed using Victor X3 plate reader (Perkin Elmer, Waltham, MA, USA) at 520 nm extinction and 590 nm emission.

### 4.6. Immunofluorescence Staining of Cryosections

mCSs were harvested by centrifugation at 50× *g* for 3 min, washed with DPBS twice, spread out in Tissue-Tek^®^ O.C.T. Compound (Sakura, Tokyo, Japan), and frozen in a nitrogen vapor. Cryosections (7 μm) of mCSs were incubated with PBS for 10 min, then fixed in a 4% formaldehyde solution for 5 min and washed with PBS three times. Some slides were permeabilized with 0.1% Triton X-100 solution for 10 min. Samples were blocked in 1% bovine serum albumin (BSA) solution supplemented with 0.3 M glycine for 1 h at room temperature following overnight incubation with primary antibodies at 4 °C. The primary antibodies include antibodies against CD31 (#553370, BD), c-Kit (MAB1356; R&D Systems, Minneapolis, MN, USA), Sca-1 (#108101, Biolegend, San Diego, CA, USA), Oct4 (ab18976, Abcam, Cambridge, UK), αSMA (ab32575; Abcam), CD105 (#105801, Biolegend), CD90 (E-AB-F1283UC, Elabscience), CD73 (sc-25603, Santa Cruz Biotechnology, Dallas, TX, USA), collagen I (A1352; Abclonal, Wuhan, China), and fibronectin (PAA037Mu01; Cloud-Clone Corp., Wuhan, China). After washing, samples were incubated with secondary antibodies conjugated to Alexa Fluor 488 or 594 (Invitrogen) for 1 h. Cell nuclei were stained with 0.1 μg/mL DAPI (Lumiprobe, Moscow, Russia) for 10 min and washed with PBS. Slides were mounted with Aqua-Poly/Mount (Polyscience, Warrington, PA, USA). Immunofluorescence was visualized with Image Exfluorer AI microscope (LCI).

### 4.7. Micro-Scale Parallel-Plate Compression Analysis of Spheroid Mechanical Properties

Spheroid mechanical properties were quantified using a micro-scale compression system MicroTester G2 (CellScale, Waterloo, ON, Canada). Individual spheroids, prepared as described in [98], were transferred to a PBS-filled reservoir of the micro tester. Each spheroid was compressed to 50% of its original diameter between a static, inflexible substrate and a dynamic cantilever beam that was provided with a fixed planar platform. Force, displacement, and real-time spheroid morphology (captured via side-view camera) were synchronously recorded. Data acquisition and analysis were performed using the CellScale software (v. 5.23).

### 4.8. Western Blotting

Spheroids were collected, washed with DPBS twice by centrifugation at 50× *g* for 3 min, and then lysed in RIPA buffer supplemented with inhibitors of proteases and phosphatases (Servicebio) on ice for 30 min. The lysates were homogenized with 31G syringes (BD) and centrifuged at 13,000× *g* for 10 min at 4 °C to remove cell debris. Protein concentration was measured using the ProteOrange kit (Lumiprobe). Samples were mixed with 6x Laemmli Sample Buffer with 4% 2-mercaptoethanol (Sigma) and heated at 95 °C for 5 min. Proteins were resolved on 8% SDS-PAGE gels using Tris-Glycine SDS running buffer and transferred to polyvinylidene difluoride (PVDF) membranes (Roche, Basel, Switzerland) using a wet-blotting system (Bio-Rad, Hercules, CA, USA) overnight at 100 mA, 4 °C. Membranes were blocked with 5% bovine serum albumin (BSA) in Tris-buffered saline with 0.05% Tween-20 (TBS-T) for 1 h. Then, membranes were incubated overnight at 4 °C with primary antibodies to collagen I (A1352, Abclonal, Wuhan, China) and β-actin (#4970, Cell Signaling Technology, Boston, MA, USA) dissolved in 1% BSA/TBS-T. After 3 washing steps with PBS-T, membranes were incubated with HRP-conjugated antibody (A16116, Invitrogen) for 1 h following washing steps. The protein bands were visualized using SuperSignal^TM^ West Pico PLUS Chemiluminiscence (Amersham Biosciences, Piscataway, NJ, USA) and were quantified using ImageJ (v. 1.54p, National Institutes of Health, Bethesda, MD, USA). Protein level was normalized to β-actin.

### 4.9. Collagen Solubility Assay

To assess collagen cross-linking, a collagen solubility assay was performed [45,99]. Spheroids were collected and washed with DPBS as described above (Section 4.8). The decellularization step was included to remove intracellular and not structural ECM. mCSs (~920,000 cells per sample) were decellularized in 0.5% Triton X-100, 20 mM NH_4_OH supplemented with protease inhibitors (300 μL per sample) for 1 h on ice. Then, spheroids were washed with DPBS twice and incubated in 0.5 M acetic acid (130 μL per sample) for 24 h at 4 °C with mild agitation to isolate the immature acid-soluble collagen (ASC) fraction. Centrifugation at 12,000× *g* for 30 min at 4 °C was performed. Supernatant (ASC) was neutralized with 10 M NaOH (final pH ~7) and frozen at −80 °C. Spheroid pellets were processed for mature collagen isolation. For that, the spheroid pellet was resuspended in 1 mg/mL pepsin (Sigma) (based on 0.5 M acetic acid solution, 130 μL per sample) and incubated for 24 h at 4 °C with mild agitation to isolate the mature pepsin-soluble collagen (PSC) fraction. After the centrifugation step, supernatant (PSC) was collected, neutralized with 10 M NaOH (final pH ~7), and frozen until use. The remaining pellet—insoluble collagen (ISC) fraction—was hydrolyzed in 1 M NaOH solution (60 μL per sample) for 1 h at 95 °C. The hydrolysates (after cooling to RT) were neutralized with glacial acetic acid (final pH ~7) and frozen until use. Blank solutions were processed identically.

Collagen I concentration in ASC and PSC fractions was measured using an ELISA kit for Collagen I (SEA571Mu, Cloud-Clone Corp.) according to the manufacturer’s recommendations. For measurement within the linear range, a 10-fold dilution of the samples was performed. Collagen I concentration in the ISC fraction was quantified by measuring hydroxyproline concentration, which constitutes ~13% of its amino acid composition [100]. An ELISA kit for Hydroxyproline (CEA621Ge, Cloud-Clone Corp.) was used.

### 4.10. Picro Sirius Red Staining

Picro Sirius Red staining protocol of fibrillar collagens was adapted from [39]. mCS cryosections were stained with heated picrosirius red solution (0.1% Direct Red 80 (Sigma-Aldrich) in saturated aqueous solution of picric acid) for 10 min, washed with acidified water (0.5% acetic acid) and running tap water, sequentially dehydrated in 70% and 96% ethanol, cleared in xylene, and mounted using Cytoseal^TM^ 60 (Epredia, Kalamazoo, MI, USA. Staining was observed using a polarized light microscope, Leica Aperio CS2 (Leica, Wetzlar, Germany).

### 4.11. Collecting mCS Condition Mediums

mCSs were washed twice and maintained in serum-free Base CS medium for 48 h. The condition media with mCSs were collected and centrifuged at 50× *g* for 3 min. The supernatants were transferred to new tubes, centrifuged at 2000× *g* for 20 min at 4 °C, and the final supernatants were frozen at −70 °C. mCSs were lysed in RIPA buffer (50 mM Tris, pH 8.0, 150 mM NaCl, 1% Triton X-100, 0.5% sodium deoxycholate, and 0.1% SDS) to measure total protein level using ProteOrange Protein Quantification Kit (Lumiprobe). The conditions mediawere normalized by total protein level.

### 4.12. Secretome Profiling

To analyze angiogenesis-related protein content, we used Proteome Profiler Mouse Angiogenesis Array Kit (ARY015, R&D Systems, Minneapolis, MN, USA). A volume of 680 μL of each mCS condition media was added to the membranes and incubated following the manufacturer’s recommendations.

### 4.13. ELISA

Vascular endothelial growth factor (VEGF) and Monocyte Chemotactic Protein 1 (MCP-1) concentrations in the mCS condition mediawere measured by ELISA Kit for Vascular Endothelial Growth Factor A (SEA143Mu, Cloud-Clone Corp.) and Mini Samples ELISA Kit for Monocyte Chemotactic Protein 1 (MEA087Mu, Cloud-Clone Corp.), respectively, in accordance with the manufacturer’s recommendations. VEGF and MCP-1 concentrations were normalized per total protein of mCSs measured with the ProteOrange kit (Lumiprobe).

### 4.14. RNA Isolation, Reverse Transcription, and Quantitative Real-Time PCR

Spheroids were collected and washed as described above (Section 4.8). mCSs (about 920,000 initially seeded cells for both assembly approaches) were lysed in RLT buffer (Qiagen, Hilden, Germany) supplemented with 2-mercaptoethanol and then homogenized with 29G syringes (BD). RNA was isolated using RNeasy Mini Kit (Qiagen, Hilden, Germany), treated with DNase I (Qiagen), and reverse-transcribed using MMLV RT kit (Eurogene, Moscow, Russia). For gene expression analysis, real-time PCR was performed using qPCRmix-HS SYBR+HighROX kit (Eurogen), as reported by us previously [101]. Primers used for PCR are listed in Table 1. Gene expression was normalized per *Actb* (β-actin).

### 4.15. Analyzing mCS Proangiogenic Properties In Vivo—Matrigel Plug Assay

mCSs formed on ULA U-well plates or PDL-coated plates were collected in centrifuge tubes, washed with DPBS twice, and transferred to 0.6 mL tubes containing 40 μL of ice-cold Matrigel^®^ Growth Factor Reduced (GFR) Basement Membrane Matrix (Corning). Matrigel loaded with mCSs at a concentration of 0.96 × 10^6^ cells per sample or 10 μL of DPBS (negative control) (four replicates per group) was transplanted subcutaneously into the mice (*n* = 6). After 14 days, mice were sacrificed, and Matrigel plugs were fresh-frozen in Tissue-Tek^®^ O.C.T. Compound (Sakura). Cryosections were stained, as described in Section 2.6, with antibodies against CD31 (#553370, BD) and von Willebrand factor (vWF, A0082, Dako, Agilent Technologies, Inc., Santa Clara, CA, USA).

### 4.16. Image Analysis

The size of mCSs was measured using NIS-Elements software (v. 5.42.01, Nikon, Yokohama, Japan). Segmentation (binary layers of spheroids) and measurements of mCS diameter were obtained with the Segment.ai artificial intelligence module and the General Analysis 3 pipeline. Transparency was measured as the mean gray value using ImageJ software (NIH).

Cell viability and death were analyzed using ImageJ software (NIH). We subtract background from fluorescence images (green and red channels separately) and measured the mean gray value for each spheroid. Fluorescence intensity was normalized per spheroid area.

CD31-, c-Kit-, and Sca-1-positive cells within mCS cryosections were counted in ImageJ using the Cell Counter plugin. Oct4-positive cells within mCS cryosections were counted in NIS-Elements software using General Analysis 3 functions to quantify nuclei with Oct4-staining inside. The number of positive cells was normalized to the total nuclei count. The content of CD105, CD90, CD73, SMA, collagen I, and fibronectin was analyzed using ImageJ. The background of fluorescence was subtracted, and the mean gray value was measured for each spheroid. Cell density within mCSs was measured as the average number of nuclei per spheroid area using ImageJ.

To detect CD31+ vessels, we quantified the CD31-positive area per Matrigel plug area in NIS-Elements software using General Analysis 3 functions.

### 4.17. Statistical Analysis

Each experiment consisted of 3–4 samples (biological replicates) per group. R version 4.3.3 was used for statistical and graphical processing of the results. Used packages include Tidyverse (v 2.0.0), rstatix (v 0.7.2), and ggpubr (v 0.6.0). Data are presented as the mean ± standard deviation. Paired two-tailed Student’s *t*-tests or multiple paired two-tailed Student’s *t*-tests were used to calculate statistical significance between two groups. One-way ANOVA was performed to compare three groups. *p*-value or adjusted *p*-value by the Bonferroni method <  0.05 was considered statistically significant.

## 5. Conclusions

In summary, our study provides the first side-by-side comparison of two CS assembly methods: ULA U-well plates against the “gold standard” method based on PDL-coated plates. This comparison reveals that the choice of technique significantly impacts the resulting spheroid phenotypic and functional properties. Both approaches reliably generated viable CSs that mimic the cardiac microenvironment, including mesenchymal cells/fibroblasts, smooth muscle, endothelial, and progenitor cells. CSs formed on PDL-coated plates were characterized by size heterogeneity, increased stiffness, compact ECM organization, and reduced EC content. Despite that, they demonstrated elevated secretion of angiogenesis-related factors and robust proangiogenic potential in vivo. In contrast, generation of mCSs on ULA U-well plates resulted in rapid formation of soft spheroids with uniform size, enhanced vascularization, elevated CD73 and fibronectin expression, and increased MCP-1 secretion. Both substrate/plate type and culture medium affect mCS morphology and gene expression. These findings are crucial for standardizing in vitro cardiac models and for selecting the appropriate spheroid assembly approach for specific downstream applications.

## Figures and Tables

**Table 1:** List of PCR primers.

Gene Symbol	Forward	Reverse
*Actb*	GGCTGTATTCCCCTCCATCG	CCAGTTGGTAACAATGCCATGT
*Col1a1*	CCGCTGGTCAAGATGGTC	CTCCAGCCTTTCCAGGTTCT
*Fn1*	GGAATGGACCTGCAAACCTA	GTAGGGCTTTTCCCAGGTCT
*Pecam1*	CACCTGTAGCCAACTTCACCAT	GCATTTCGCACACCTGGATC
*Vegf*	GGAGACTCTTCGAGGAGCACTT	GGCGATTTAGCAGCAGATATAAGAA
*Mmp2*	ACCTGAACACTTTCTATGGCTG	CTTCCGCATGGTCTCGATG

## Data Availability

The original contributions presented in this study are included in the article/Supplementary Materials. Further inquiries can be directed to the corresponding author.

## Supplementary Materials

The following supporting information can be downloaded at: https://www.mdpi.com/article/10.3390/ijms262412025/s1.

## Abbreviations

The following abbreviations are used in this manuscript:

CSs	Cardiospheres
PDL	Poly-D-lysine
ULA	Ultra-low attachment
ECM	Extracellular matrix
EDCs	Explant-derived cells
CDCs	Cardiosphere-derived cells
ECs	Endothelial cells
