# Supplementary figures and images for: The 3D World of Spheroids: Searching for an Optimal Method of Fabricating Pro-Reparative Cardiospheres
Source: Int J Mol Sci. 2025 Dec 13;26(24):12025. doi: 10.3390/ijms262412025 (PMC12733164; doi:10.3390/ijms262412025)

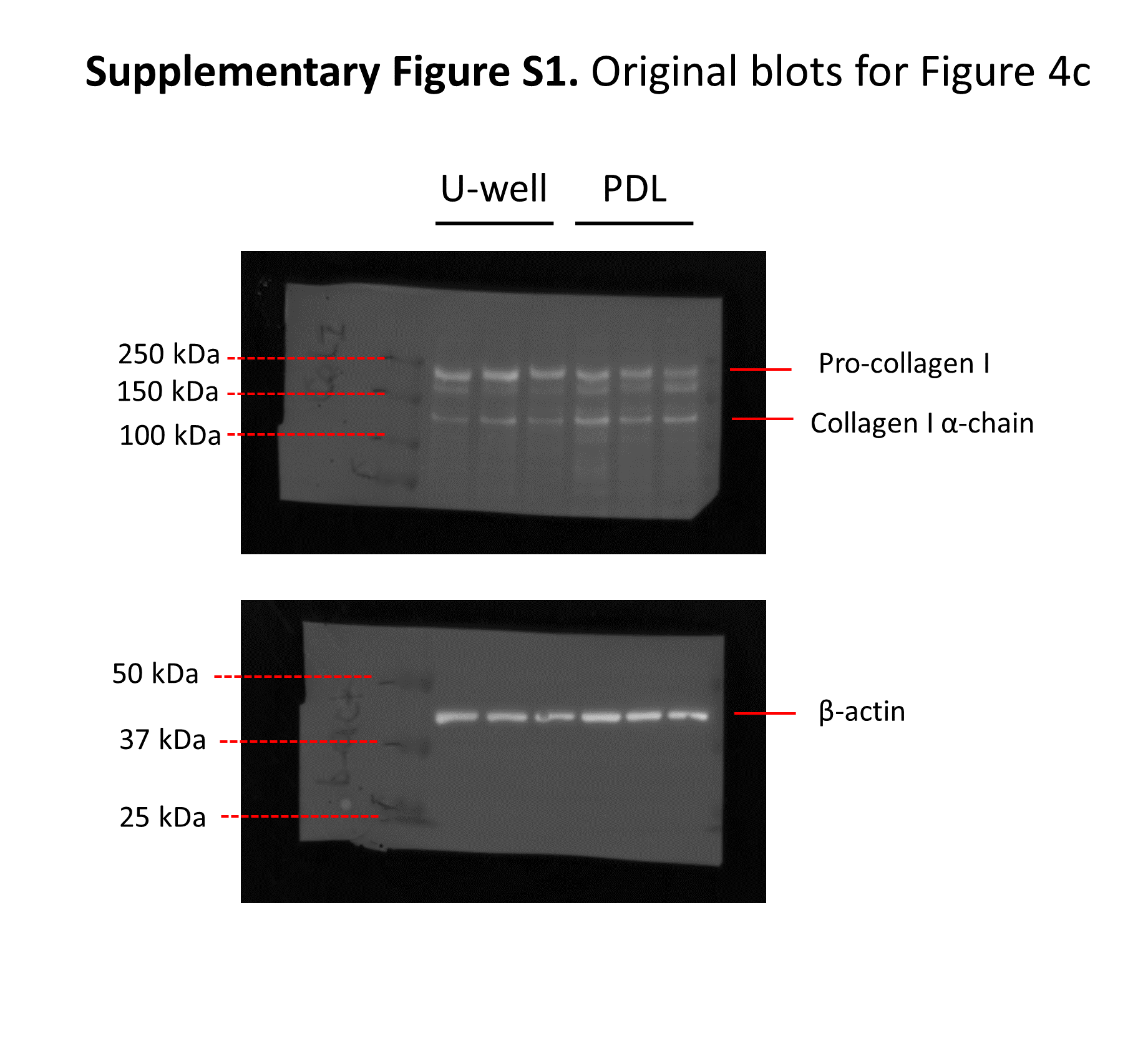

Supplement: Supplementary file 1 [file ijms-26-12025-s001.zip › Supplementary_Figure_S1.png]

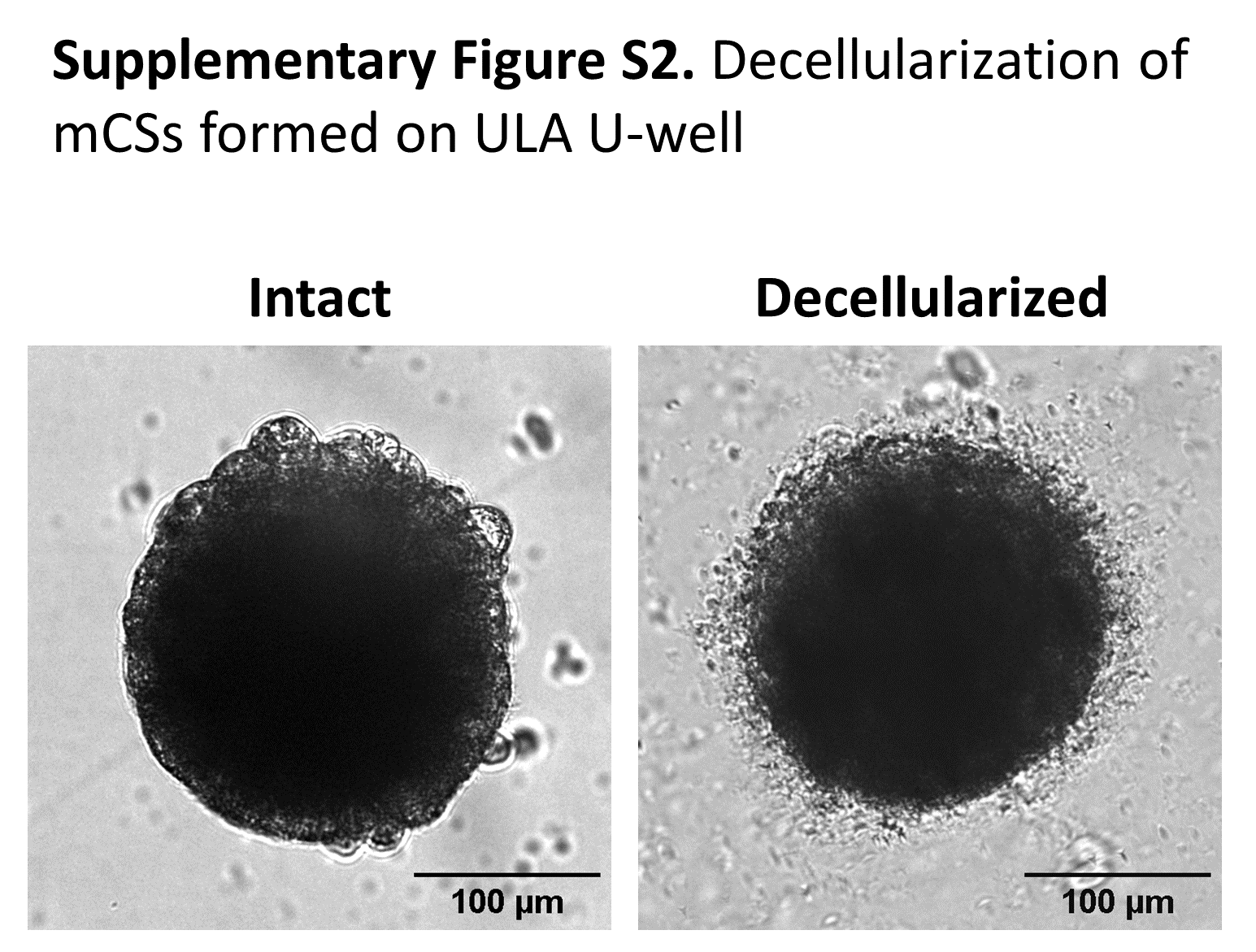

Supplement: Supplementary file 1 [file ijms-26-12025-s001.zip › Supplementary_Figure_S2.png]
